# Social network typologies moderate the association of loneliness with depressive symptomatology in middle-aged and older adults

**DOI:** 10.3389/fpsyt.2023.1141370

**Published:** 2023-05-05

**Authors:** Huiyoung Shin, Chaerim Park

**Affiliations:** Department of Psychology, Jeonbuk National University, Jeonju, Republic of Korea

**Keywords:** network typologies, loneliness, depressive symptomatology, middle-aged, older adults

## Abstract

**Background:**

Depression remains among the most prevalent mental disorders, and it severely affects daily functioning and quality of life. There has been extensive research reporting on the impact of social relationships on depression, but much of this research has only considered isolated aspects of relationships. This study derived social network types based on the multiple components of social relationships, and then investigated their effects on depressive symptoms.

**Methods:**

Using samples of 620 adults (*M_age_* = 53.52), Latent Profile Analysis (LPA) was conducted to uncover network types based on the structural (network size, contact frequency, marital status, and social engagement), functional (levels of support and conflict), and qualitative (satisfaction with relationships) aspects of social relationships. Multiple regressions were used to test if distinct network types directly impact on depressive symptoms and whether network types moderate the association of loneliness (perceived social isolation) with depressive symptoms.

**Results:**

LPA identified four distinct network types (*diverse, family-focused*, *friend-focused*, and *restricted*) and there were significant differences in depressive symptoms among four network types. Analysis using the BCH method showed that individuals in the *restricted* network type had the highest depressive symptoms, followed in order by individuals in the *friend-focused*, *family-focused*, and *diverse* network types. Regression results further indicated that an individual’s network network type membership was significantly associated with depressive symptoms, and being in the *diverse* and *family-focused* network types alleviated the negative effect of loneliness on depressive symptoms.

**Conclusion:**

The results suggest that both quantitative and qualitative aspects of social relationships are important in buffering against the adverse effect of loneliness on depressive symptoms. These findings underscore the utility of taking a multi-dimensional approach to uncover heterogeneity in the social networks of adults and their implications on depression.

## Introduction

Depression is one of the most prominent and prevalent mental disorders worldwide, and it severely affects individuals’ daily functioning and quality of life and contributes substantially to global disability (1). A recent report by the World Health Organization estimated that the number of people suffering from major depression is 322 million and has increased by 18.4% worldwide between 2005 and 2015 (2). The main symptoms of depression include a lack of interest in life activities, feelings of worthlessness or inappropriate guilt, fatigue, insomnia or hypersomnia, psychomotor agitation or retardation, and recurrent thoughts of death or suicidal ideation (3). Aside from being a debilitating mental disorder in and of itself, depression increases the risk for functional impairment such as daily living and mobility disability along with cognitive impairment such as Alzheimer’s disease (4, 5). Patients with major depression are also at risks of developing cardiovascular disease and increased morbidity and mortality (1, 6). One of the most urgent aspects of depression is that patients with major depression are more likely to commit suicide. The association of depression and suicide has been well established in the literature (7–9), and it is reported that 15% of clinically depressed patients die by suicide (10, 11). Moreover, suicidal ideation and consummated suicide is comparatively high among older adults (12–14), and those who attempt suicide are more likely to be widow(er)s, live alone, lack a confidant, have poor self-rated health, and experience stressful life events such as financial or interpersonal discord (15, 16).

As mentioned above, a loss of (or a lack of) confidants or close social contacts has–along with various other social relationship variables–been shown to be a significant predictor of depression and its adverse health implications (17). In fact, there is ample evidence indicating that depression is partly a social mental disorder, with reduced social connectedness implicated as both a cause and symptom, and it is therefore considered a target for treatment (18). Marked decreases in social connections typically emerge prior to the development of depressive symptomatology, and social isolation is reported to be a strong risk factor for the development and recurrence of depression (19, 20). For instance, perceived social isolation has been shown to be a significant precursor of depressive symptomatology while controlling for demographic characteristics, personality, stress, and physical health (21), and a lack of supportive interactions has been shown to predict suicidal ideation and attempt (22), as well as consummated suicide (23). Interpersonal conflict has also been shown to be the most robust stressor for daily fluctuations of negative mood, with accumulated and escalating effects when it continues over a few days (24–26). Empirical evidence indicates that individuals are particularly sensitive to social stressors relative to other stressful life events, and depression is often triggered by specific negative social life event such as the loss of a loved one, family conflict, or relationship breakdown (27–29). As a corollary of this, social connectedness has been reported to be critical in alleviating depressive symptoms and come to be considered a core component of depression treatment (30–32).

Indeed, the characteristics of individuals’ social relationships are key determinants of mental health outcomes, including depression. Those with supportive social networks and positive social interactions with multiple sources show better physical and mental health outcomes (33, 34). Although there has been extensive research examining the impact of social relationships on depression, much research has considered isolated aspects of individuals’ social relationships such as the size of their networks, contact frequency, and perceived or received support and their implications, and the results have been inconsistent (17). Such inconsistent findings could be attributed to the fact that most research has taken a linear approach to investigate the effects of a single aspect or just a few aspects of social relationships (35). Although such a variable-centered linear approach is informative, it may not capture the multidimensional aspects that characterize individuals’ social networks and may overlook the variance in supportive or conflicting interactions among heterogeneous groups of individuals (36, 37). Taking a person-centered approach, research on social network typology has shown that the unique make-up of network characteristics and the configuration of different relationships in social networks are predictive of individuals’ physical and mental health (38–42).

According to the social convoy model (43), an individual’s social convoy refers to a group or social network of people with whom a person is linked. An individual’s social convoy is shaped by personal (e.g., age, gender, education) and situational (e.g., roles, expectation, resources, events, historical context) factors and dynamic in nature that moves through the entire life course (44). The social convoy model emphasizes that social relationships are multidimensional (45). For instance, it specifies that social convoys consist of different dimensions of *structure* (describing the size and composition of one’s networks, marital status, contact frequency, and participation in social activities), *function* (describing features of actual and perceived interactions), and *quality* (describing individuals’ subjective evaluations of interactions) of social relationships (39, 46). Each of these components shapes the social context in a different way across the life course, by directly influencing mental health and/or by indirectly providing compensatory resources during times of stress (46).

**Figure 1:**
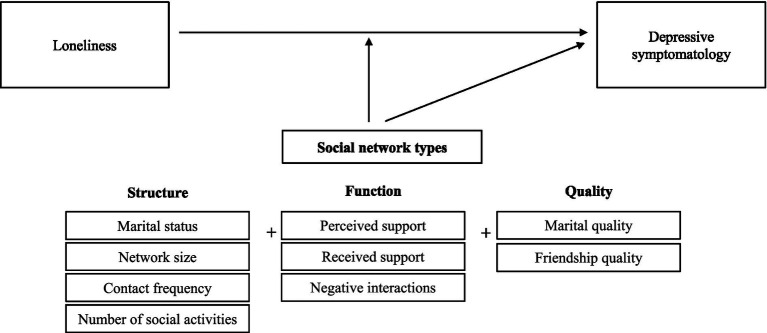
Conceptual model.

Despite the theoretical support for the multidimensional nature of social relationships, only a handful of studies have considered comprehensive dimensions of social networks (39, 47), and most previous research has primarily focused on the structural (38, 48–50) or functional aspects of social networks (35, 51–53). Further, to our knowledge no prior research has empirically investigated the possible mechanisms by which network types moderate the association of depressive symptomatology with its precursors. Therefore, in this study, we consider structural, functional, and qualitative components of social networks in concert to identify distinct network types and then examine how heterotypic network types that reflect the varied patterns of network characteristics are differentially associated with depressive symptomatology and moderate the association of loneliness (also termed perceived social isolation)–which is one of the most potent risk factors of depression–with depressive symptomatology in a sample of South Korean adults, all while controlling for various demographic indicators (age, gender, marital status, education, income, and self-rated health). Specifically, we investigated the following research questions: (a) What network types are typically found based on network structural, functional, and qualitative aspects among middle-aged and older adults? (b) Is membership in a particular network type associated with depressive symptomatology after controlling for demographic and covariate variables? (c) Does membership in a particular network type moderate the association of loneliness (perceived social isolation) with depressive symptomatology? (see Figure 1 for our conceptual research model).

## Materials and methods

### Procedure

After receiving an approval from the university’s IRB, we recruited participants from an online research participant system, which maintains a panel of 1,663,404 South Korean adults across varying age, education, and demographic characteristics. We used stratified probability sampling to obtain a representative sample of different age groups and geographical regions. We invited the pool of participants to take an online survey and provided the information about the current research from 29 January to 5 February 2021. Informed consent was obtained from each participant before their participation. They were told that participation of the survey was voluntary and that the collected information would be kept confidential. Participants completed a survey that took about 30 min and they were given gift certificates upon completion.

### Participants

The original sample comprised 1,033 adults (49.85% male) representing each decade of adult life span (i.e., 20–29 years, *n* = 210; 30–39 years, *n* = 203; 40–49 years, *n* = 204; 50–59 years, *n* = 209; 60–69 years, *n* = 207). The number of participants were also well represented by the geographical region (i.e., 5–7% for 17 different municipalities). Because the target of this study was middle-aged and older adults, we only used participants who were aged 40 years and older. The final sample was 620 adults (50.81% male) aged between 40 and 69 years old (*M_age_* = 53.52, *SD* = 8.34). About 23% of the participants had less than a high school diploma, the majority (65%) had a college degree, and 12% had a degree higher than a college degree. More than one quarter (31%) of the participants reported having an annual household income of less than $20,000, and 41% reported having an annual household income of more than $40,000. The majority of the participants were married (79%) and had at least one child (84%). We provided more detailed demographic information in Appendix. There were no missing data, and all participants responded to items on network characteristics, loneliness, and depressive symptoms.

### Measures

We used information on network characteristics (structure, function, and quality) to identify distinct network types, and we set depressive symptomatology as an outcome. Information on demographic variables and self-rated health were used as covariates while loneliness was used as a predictor and a moderator (see Figure 1). The descriptive information and intercorrelations for all variables used in the current study are provided in Appendix.

#### Social network characteristics

We assessed seven *structural* social network variables using the Berkman-Syme Social Network Index (54). It is a validated measure that assesses one’s degree of social integration and sociability including marital status, size and frequency of contact with family and friends, and number of social activities. We dichotomized marital status into not married (widowed, divorced, or not married; 0) or married (1). We assessed the size and contact frequency with family (defined as spouse, children, siblings, and parents) and friends that individuals had spent time with in the past 4 weeks. We included the total number of children and the total number of social activities in which the individuals currently participated (e.g., gym classes, community or church service).

To assess the *functional* aspect, we considered 11 social network variables of perceived and received support and negative interactions (i.e., social conflict). Perceived support was measured using the Multidimensional Scale of Perceived Social Support (55), which consists of 12 items assessing perceived support from family, friends, and close others. A sample item is “My family is willing to help me make decisions.” Each item was scored from 1 (*not at all true*) to 5 *(very true).* The average score was calculated for each subscale, with higher scores indicating greater perceived support. The Cronbach’s α scores for perceived support from family, friends, and close others were 0.91, 0.93, and 0.91, respectively. Received support and negative interactions were measured using the Positive and Negative Social Support Scale (56), which consists of 28 items measuring support and conflict for the four relationships (spouse, friend, child, and sibling). Sample items are “How much do you rely on them when you have a serious problem?” for support and “How much do they criticize you?” for conflict. Each item was scored from 1 (*not at all true*) to 5 (*very true*). The average score was calculated for each subscale, with higher scores indicating greater received support and conflict. For the subscales of spouse, friend, child, and sibling, the Cronbach’s α scores were 0.86, 0.83, 0.82 and 0.88 for received support and 0.83, 0.86, 0.83, and 0.90 for conflict, respectively.

To assess the *qualitative* aspect, we included two variables of relationship quality: marital quality and friendship quality. Marital quality was measured using the Quality Marriage Index (57), which consists of six items assessing the global quality of one’s marriage. A sample item is “My relationship with my spouse is very stable.” Each Item was scored from 1 (*not at all true*) to 5 (*very true*), with higher scores indicating greater marital quality. In the present study, the Cronbach’s α of this scale was 0.95. Friendship quality was measured using Rose’s (58) adapted version of Friendship Quality Questionnaire (59). Original measure consists of 19 items that assess validation and caring, conflict resolution, conflict and betrayal, help and guidance, companionship and recreation, and intimate exchange. We used 12 items that are applicable to adults and reworded some of the items for age appropriateness. A sample item is “I am satisfied with my relationship with my friend.” Each item was scored from 1 (*not at all true*) to 5 (*very true*), with higher scores indicating greater friendship quality. In the present study, the Cronbach’s α of this scale was 0.90.

#### Demographic variables and self-rated health

The Demographic variables considered in this study were age, gender, marital status, retirement status, education, and income. Age in years was used as a continuous variable. Gender as well as marital and retirement status were all dichotomized (0 = male, 1 = female; 0 = not married, 1 = married; 0 = not retired, 1 = retired). Education was classified from 1 (≤ elementary school) *to* 5 (graduate school). Income was classified from 1 (≤ $10,000) *to* 5 (5 ≥ $40,000). Self-rated health was classified from (1 to 5), with 5 being the highest level, was used as a continuous variable.

#### Loneliness

Loneliness was measured using the UCLA Loneliness Scale (60). Participants rated the extent to which they agreed with each of the 20 statements using a 5-point scale (1 = *not at all true* to 5 = *very true*). A sample statement includes “I feel isolated from others.” The mean score was calculated, with higher scores indicating higher levels of loneliness. In the present study, the Cronbach’s α of this scale was 0.94.

#### Depressive symptomatology

Depressive symptomatology was measured using the 20-item Center for Epidemiological Studies-Depression (CES-D) Scale (61), which assesses depressive symptoms experienced during the past week. Sample statements included “I felt I could not shake off the blues” and “I talked less than usual.” Each item was scored from 0 (*rarely*) *to* 3 (*most or the time*), and scores were summed to create a scale that ranged from 0 to 60, with higher scores indicating higher levels of depressive symptoms. In the present study, the Cronbach’s α of this scale was 0.94.

### Analytic strategy

All statistical analyses were conducted using SPSS 25.0 and Mplus 8.6. We used SPSS 25.0 for descriptive statistics and multiple regression analyses. We used Mplus 8.6 to conduct Latent Profile Analyses (LPA) to uncover social network types based on the structural, functional, and qualitative aspects of individuals’ social relationships. The raw score of all indicators were converted to a z-score for the analyses, with a z-score of zero representing the overall sample mean. LPA empirically determines distinct latent profiles into which participants with similar characteristics can be assigned, and then provides estimate mean scores for each of the profiles (62). Derived profiles can be also incorporated into the LPA model to build a linear regression mixture model to investigate the relationship between latent profiles and distal outcomes (BCH method) (63). The BCH method is considered to be more robust because it is similar to a standard ANOVA (64) and to substantially outperform previous method (e.g., Lanza’s method and the 3-step method) in that it avoids shifts in latent profiles in the final stage (65).^1^ The BCH method evaluates the mean of a continuous distal outcomes across different profiles using the approach of Bakk and Vermunt (2016), which is recommended over the *post hoc* approach because it allows for the uncertainty of profile assignment to remain in the model (63, 64). In this study, we used LPA with the BCH method to identify distinct network types and to compare significant differences across network types (latent profiles) in the levels of depressive symptomatology.

A series of models with progressively increasing number of profiles from two to five were estimated and compared to determine the most optimal solution for the data. After running four models with different numbers of profiles, we compared fit indices across profiles based on lower Akaike Information Criterion (AIC), lower Bayesian Information Criterion (BIC), lower sample-size adjusted BIC (SABIC) values, higher entropy values, and significant Lo–Mendell–Rubin adjusted likelihood ratio test (LMR-LRT) (68). We provided multiple fit indices and profile distributions of each model in Appendix. For AIC, BIC, and SABIC, solutions with a larger number of profiles provided a better fit. The five-profile solution included a profile with less than 5% of the sample. Because solutions with a sample less than 5% indicate that too many profiles have been derived (69), we did not consider this profile solution for our final model. Based on the theoretical appropriateness and interpretability (70), the four-profile model solution was determined as our final model. The entropy of the final model was 0.89, indicating that 89% of participants were correctly classified.

Then, we examined if the levels of depressive symptoms in one network type is significantly different from those in other network types using the BCH method suggested by Asparouhov and Muthen (63). Based on the derived network types, we conducted multiple regression analyses. First, to examine the direct impact of network types on depressive symptomatology, we used the main effect model, which included the dependent variable (depressive symptomatology), independent variable (membership in network types), demographic variables, and covariates. Next, to examine the moderating role of network types in the association of loneliness with depressive symptomatology, we used the interaction effect model, which additionally included the interaction terms between loneliness and network types.

## Results

### Latent profile analyses

**Figure 2:**
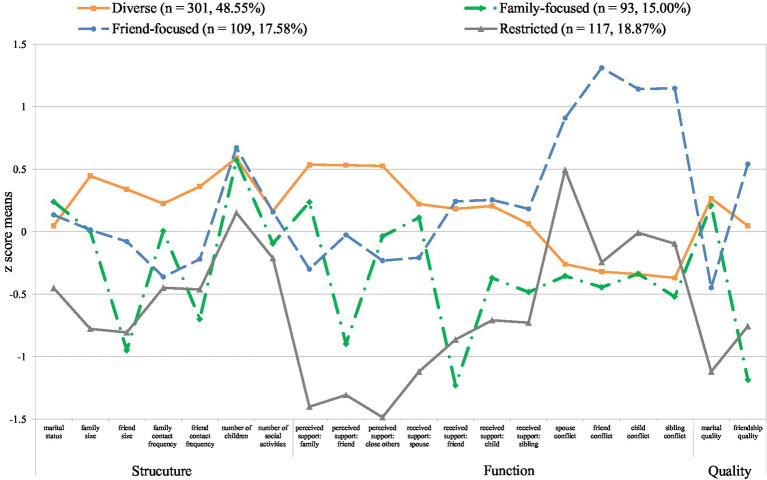
Social network types based on network structure, function, and quality (*N* = 620).

Social network types were identified based on 20 network indicators capturing the structure, function, and quality of social relationships. Figure 2 presents the final four profiles (i.e., social network types): *diverse*, *family-focused, friend-focused*, and *restricted.* We also provide detailed information about the group means and proportions for entire social network indicators by the four derived network types in Table 1. The *diverse* network type (*n* = 301, 48.55%) consisted of individuals with the highest network size and contact frequency with family and friends with which they had spent time within the past 4 weeks, and who had above-average engagement in social activities. They reported the highest levels of perceived and received support, along with below-average levels of conflict. They also reported above-average levels of marital and friendship quality. Individuals in the *family-focused* network type (*n* = 93, 15.00%) had an average network size and contact frequency with family, but below-average contact frequency with friends. They reported above-average levels of both perceived and received support and the lowest levels of conflict from family, while they reported below-average levels of perceived and received support from friends. They also reported the highest marital quality, but the lowest friendship quality. Individuals in the *friend-focused* network type (*n* = 109, 17.58%) had an average network size of family and friends, and the highest engagement in social activities. They reported above-average received support from friends, children, and siblings, but below-average perceived support from family and close others. Interestingly, they rated their social relationships as highly negative; they reported the highest levels of conflicts with family and friends. They also reported low marital quality whereas they reported the highest friendship quality. The *restricted* network type (*n* = 117, 18.87%) consisted of individuals who were unmarried or with relatively small social networks; that is, infrequent contacts with family and friends, and a small proportion of close others. They reported low levels of perceived and received support, above-average spousal conflict, and the lowest marital quality.

**Table 1:** Group means and proportion for entire social network indicators for different social network types.

Variables	Diverse	Family-focused	Friend-focused	Restricted
	(*n* = 301, 48.55%)	(*n* = 93, 15.00%)	(*n* = 109, 17.58%)	(*n* = 117, 18.87%)
**Structure**				
Married (proportion)	0.81	0.88	0.85	0.60
Family size	3.75	3.38	3.35	**2.59**
Friend size	**3.18**	**1.76**	2.73	**1.95**
Family contact frequency	**4.21**	**3.82**	3.21	3.15
Friend contact frequency	3.50	**1.85**	2.61	2.23
Number of children	**1.64**	**1.60**	**1.76**	1.21
Number of social activities	1.62	1.44	1.65	1.31
**Function**				
Perceived support: family	**4.16**	3.92	3.37	**2.35**
Perceived support: friend	**3.86**	**2.43**	3.34	**2.09**
Perceived support: close others	**4.06**	3.55	3.36	**2.17**
Received support: spouse	3.76	3.67	3.36	**2.49**
Received support: friend	3.31	**2.06**	3.37	**2.41**
Received support: child	3.04	2.48	3.10	**2.17**
Received support: sibling	2.87	2.30	2.99	**2.07**
Spouse conflict	2.13	2.01	**3.21**	2.82
Friend conflict	1.54	1.47	**2.99**	1.61
Child conflict	1.70	1.69	**3.01**	2.00
Sibling conflict	1.50	**1.38**	**2.87**	1.74
**Quality**				
Marital quality	3.94	3.91	3.22	**2.55**
Friendship quality	2.78	**1.89**	**3.15**	**2.22**

Boldfaced numbers indicate defining peaks of the profile types (specifically, approximately 0.5 or >0.5 SD above or below the sample mean). The ranges are as follows: family and friend size, 1–6; family and friend contact frequency, 1–6; number of children, 0–4; number of social activities, 1–6; perceived support, 1–5; received support, 1–5; conflict, 1–5; marital quality, 1–5; friendship quality, 1–5.

**Figure 3:**
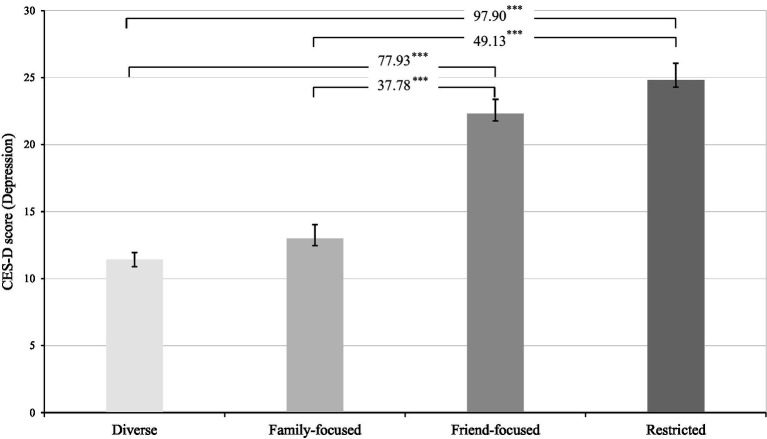
Significant differences in the mean levels of depressive symptomatology across four social network types. Analysis was conducted with the BCH procedure; Differences in mean CES-D scores between network types were significant at *p* < 0.001. Numbers indicate χ^2^ statistics values.

As provided in Table 2, the largest network type was the *diverse*, which comprised the second largest proportion of older adults (35.88%) and had 80.73% married individuals. Members of the *diverse* network type reported the best self-rated health (*mean* = 3.26) and the lowest loneliness (*mean* = 2.11) along with the lowest depressive symptomatology (*mean* = 11.42). The *family-focused* network type was characterized by the highest proportion of older adults (38.71%), as well as better self-rated health (*mean* = 3.16) and lower loneliness (*mean* = 2.78) and depressive symptomatology (*mean* = 13.00) relative to the *friend-focused* and *restricted* network types. The *friend-focused* network type was characterized by the smallest proportion of older adults (21.10%), better self-rated health (*mean* = 3.16), and lower loneliness (*mean* = 2.81) and depressive symptomatology (*mean* = 22.30) relative to the *restricted* network type (but worse loneliness and depressive symptomatology relative to the diverse and family-focused network types). The *restricted* network type consisted of 59.83% married individuals. This group had the lowest levels of income (*mean* = 2.99) and self-rated health (*mean* = 2.87), and the highest levels of loneliness (*mean* = 3.29) and depressive symptomatology (*mean* = 24.82). All of these differences were statistically significant (see Table 2; Figure 3).

**Table 2:** Differences in demographic variables, covariates, and depressive symptomatology between four social network types.

Variables	1	2	3	4	Statistics
	Diverse (*n* = 301, 48.55%)	Family-focused (*n* = 93, 15.00%)	Friend-focused (*n* = 109, 17.58%)	Restricted (*n* = 117, 18.87%)	
Age *M* (*SD*)	53.84_a_ (8.46)	55.32_a_ (7.80)	51.66_b_ (7.91)	53.03_a_ (8.56)	*F* (3, 616) = 3.58^*^
Age group (%)	108 (35.88%)	36 (38.71%)	23 (21.10%)	40 (34.19%)	$\;\chi^{2}$ (3) = 9.46^*^
Gender (%)	151 (50.17%)	43 (46.24%)	45 (41.28%)	66 (56.41%)	$\;\chi^{2}$ (3) = 5.61
Married (%)	243 (80.73%)	82 (88.17%)	93 (85.32%)	70 (59.83%)	$\;\chi^{2}$ (3) = 33.44^***^
Retirement (%)	80 (26.57%)	31 (33.33%)	20 (18.35%)	31 (26.50%)	$\;\chi^{2}$ (3) = 5.96
Child (%)	265 (88.04%)	79 (84.95%)	95 (87.16%)	81 (69.23%)	$\;\chi^{2}$ (3) = 23.35^***^
Sibling (%)	292 (97.01%)	88 (94.62%)	103 (94.50%)	111 (94.87%)	$\;\chi^{2}$ (3) = 2.13
Education *M* (*SD*)	3.89 (0.62)	3.92 (0.59)	3.86 (0.55)	3.77 (0.74)	*F* (3, 616) = 1.31
Income *M* (*SD*)	3.51_a_ (1.64)	3.47_a_ (1.60)	3.60_a_ (1.62)	2.99_b_ (1.48)	*F* (3, 616) = 3.61^*^
Self-rated health *M* (*SD*)	3.26_a_ (0.72)	3.16_a_ (0.77)	3.16_a_ (0.74)	2.87_b_ (0.79)	*F* (3, 616) = 7.73^***^
Loneliness *M* (*SD*)	2.11_a_ (0.03)	2.78_b,c_ (0.07)	2.81_b,c_ (0.05)	3.29_b,d_ (0.06)	*F* (3, 616) = 135.34^***^
Depression *M* (*SD*)	11.42_a_ (0.53)	13.00_a_ (1.04)	22.30_b_ (1.08)	24.82_c_ (1.25)	*F* (3, 616) = 60.17^***^

M, mean; SD, standard deviation. The scale ranges are as follows: age = 40–69; age group, 1 = older adults; gender, 1 = female; married, 1 = married; retirement, 1 = retired; child, 1 = have children; sibling, 1 = have siblings; education, 1 ≤ elementary school, 2 = middle school, 3 = high school, 4 = some college, 5 = graduate school; income, 1 ≤ $10,000, 2 = $10,000–$20,000, 3 = $20,000–$30,000, 4 = $30,000–$40,000, 5 ≥ $40,000; self-rated health, 1–5; loneliness, 1–5; depressive symptomatology, 0–3. Means in the same row that do not share subscripts significantly differ at *p* < 0.05 in the Bonferroni comparison; ^*^p < 0.05. ^**^p < 0.01. ^***^p < 0.001.

### Multiple regression analyses

**Figure 4:**
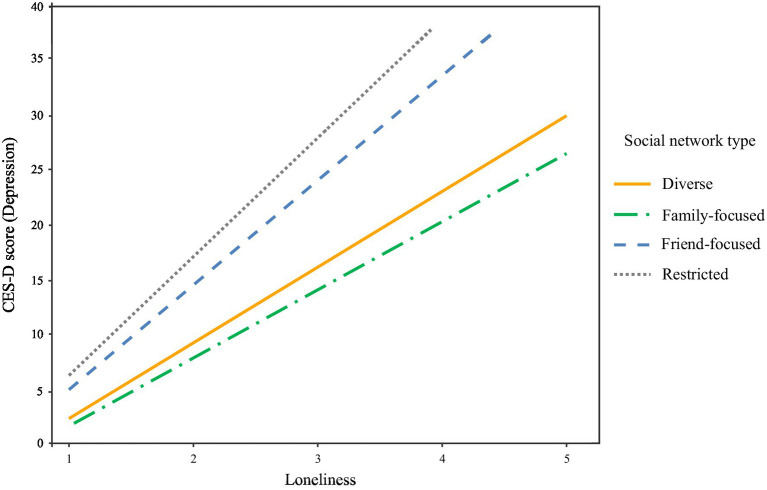
Interaction effect of loneliness and social network types on depressive symptomatology.

For a preliminary analysis, we evaluated the normality assumption of depressive symptomatology. It is expected that skewness values smaller than 3.0 and kurtosis values smaller than 8.0 indicate the acceptable normality (71). In this study, skewness value was 1.07 and kurtosis value was 0.96, which were much below the acceptable threshold. Thus, univariate normality of depressive symptomatology was confirmed. Table 3 presents the results of the multiple regression analyses. The main effect model demonstrated that, when controlling for individuals’ demographic characteristics (i.e., age, gender, marital status, retirement status, education, income, and self-rated health), an individual’s network type membership was significantly associated with their depressive symptomatology. Specifically, compared to being in the restricted network type, being in the diverse (*β* = −0.12, *p* < 0.05) and family-focused network type (*β* = −0.20, *p* < 0.001) was associated with lower levels of depressive symptomatology and being in the friend-focused network type (*β* = 0.10, *p* < 0.05) was associated with higher levels of depressive symptomatology. Loneliness was a significant predictor of depressive symptomatology (*β* = 0.48, *p* < 0.001), and those who were older (*β* = −0.11, *p* < 0.01) and had better self-rated health (*β* = −0.20, *p* < 0.001) reported lower depressive symptomatology. Our interaction effect model further demonstrated that network types moderated the association of loneliness with depressive symptomatology. After controlling for the significant effect of loneliness on depressive symptomatology (*β* = 0.66, *p* < 0.001), being in the diverse and family-focused network types were shown to alleviate the negative effect of loneliness on depressive symptomatology (see Figure 4). The inclusion of these interaction effects accounted for a significant additional variance of 0.83% in depressive symptomatology over and above the variance accounted for by network types, loneliness, demographic variables, and covariates (∆*F* (3, 605) = 3.18, *p* < 0.05).

**Table 3:** Regression coefficients predicting depressive symptomatology.

	Main effect model				Interaction effect model			
	*β*	95% CI		*p*-value	*β*	95% CI		*p*-value
^a^ **Social network type**								
Diverse	−0.12^*^	−0.21	−0.02	< 0.05	−0.04	−0.15	0.07	0.46
Family-focused	−0.20^***^	−0.28	−0.13	< 0.001	−0.12^*^	−0.21	−0.02	< 0.05
Friend-focused	0.10^*^	0.02	0.18	< 0.05	0.17^**^	0.07	0.26	< 0.01
Loneliness	0.48^***^	0.41	0.56	< 0.001	0.66^***^	0.52	0.81	< 0.001
**Loneliness** × **Network type**								
Loneliness × Diverse					−0.15^**^	−0.26	−0.04	< 0.01
Loneliness × Family-focused					−0.12^**^	−0.20	−0.03	< 0.01
Loneliness × Friend-focused					−0.04	−0.11	0.03	0.30
Age	−0.11^**^	−0.18	−0.04	< 0.01	−0.11^**^	−0.18	−0.04	< 0.01
^b^Gender	0.01	−0.05	0.08	0.64	0.01	−0.05	0.07	0.79
^c^Marital status	0.01	−0.05	0.07	0.80	0.00	−0.06	0.06	0.99
^d^Retirement status	0.06	−0.01	0.13	0.08	0.06	−0.01	0.12	0.11
Education	−0.02	−0.08	0.05	0.61	−0.02	−0.08	0.04	0.54
Income	−0.06	−0.12	0.01	0.07	−0.06	−0.12	0.01	0.07
Self-rated health	−0.20^***^	−0.27	−0.14	< 0.001	−0.21^***^	−0.27	−0.15	< 0.001
*R*^2^ (Adjusted *R*^2^)	0.46 (0.45)				0.47 (0.46)			
*F*	47.72^***^				38.58^***^			

*β* = standardized coefficient; 95% CI, 95% confidence interval; ^a^Restricted network type was specified as the reference group; ^b^Gender is coded as 1 = female; ^c^Marital status is coded as 1 = married; ^d^Retirement is coded as 1 = retired. The scale ranges are as follows: age = 40–69; education, 1 ≤ elementary school, 2 = middle school, 3 = high school, 4 = some college, 5 = graduate school income, 1 ≤ $10,000, 2 = $10,000–$20,000, 3 = $20,000–$30,000, 4 = $30,000–$40,000, 5 ≥ $40,000; self-rated health, 1–5; loneliness, 1–5; depressive symptomatology, 0–3; ^*^p < 0.05. ^**^p < 0.01. ^***^p < 0.001.

## Discussion

In the current study, we examined how heterotypic network types that reflect varied patterns of network characteristics are differentially associated with depressive symptoms and moderate the association of loneliness with depressive symptoms among middle-aged and older adults in South Korea. The present work used a person-oriented latent profile analysis, which provides a way to consider an individual’s social relationships in a naturally complex and aggregated state, thus providing a more holistic depiction of multidimensional social networks that could more accurately predict depressive symptoms. This approach is consistent with many theories on social relations that emphasize the importance of considering multiple sources and aspects of social relationships simultaneously and their functional specificity [the social convoy model (42); the functional specificity theory (72, 73)]. Overall, our findings underscore the importance of considering more varied assessments of social relationships to uncover heterogeneity in the social networks of adults and their implications on depressive symptomatology.

Using a profile-based approach, we uncovered four distinct social network types reflecting different configurations of structural, functional, and qualitative attributes of social relationships. The four derived network types were broadly consistent with those uncovered in previous network typology research (38, 39, 74, 75), and they had significant direct and indirect effects on individuals’ depressive symptomatology. Specifically, participants in the *diverse* and *family-focused* network types reported lower depressive symptoms than those in the *friend-focused* and *restricted* network types, to an extent far beyond that of several key demographic variables and covariates. Our findings also revealed that individuals in the *diverse* network type reported the lowest depressive symptomatology (mean score of 11.42 on the CES-D), whereas individuals in the *restricted* network type reported the highest depressive symptomatology (mean score of 24.82 on the CES-D). This finding could be related to the salient impact of multiple relationship sources and broader network structural aspects in promoting social connectedness and social integration as well as role fulfillment and a sense of purpose, all of which could translate into better mental health and lower depressive symptoms (45, 72, 73, 76).

Our findings also revealed that the presence of negative interactions (i.e., conflict) has particularly detrimental effects in depressive symptoms. Consistent with prior evidence indicating that conflict and strain have more potent effects on mental health than social support (25, 26, 77, 78), individuals in the *friend-focused* network type (characterized as having average network size, the highest engagement in social activities, and above-average received support, but the highest levels of conflicts with family and friends) reported the second highest (almost comparable to the highest score found in the *restricted* network type) depressive symptomatology (mean score of 22.30 on the CES-D). It can therefore be assumed that, although social support occurring across multiple social relationships and having many social roles in the family and the community can have additive benefits, conflicting social interactions themselves can dampen such beneficial effects of multiple support and roles on depressive symptomatology.

In addition to the direct effect, being in the *diverse* and *family-focused* network types was found to alleviate the adverse effect of loneliness on depressive symptoms. This finding provides qualified support for the protective roles of social network features. The nature of the interactive effects suggests that those who perceive themselves as being socially isolated but who have access to multiple relationship sources (i.e., *diverse* type) or high levels of support (i.e., *family-focused* type) may be better able to cope with or recover from depressive symptoms than those isolated adults who do not have such access. The buffering role of network features suggested by these findings is consistent with theoretical and empirical evidence on the processes linking social resources to mental health (79, 80). These results indicate that, in addition to the support provided by strong social ties (i.e., close social network members such as spouses or close friends), weak social ties (i.e., peripheral social network members such as neighbors and colleagues) can also provide important resources for coping with challenges by providing empathic understanding, coping encouragement, and needed support during times of stress. The effectiveness of such support would be enhanced when the social network members who provide this support are similar and have experienced comparable stressors. Similar others are better able to provide cognitive appraisal or informational support because they are comparable to the distressed individual in terms of social and personal characteristics, attitudes, or stress experiences (81, 82). Having more diverse social networks predictably increase the opportunity to encounter similar others who can afford different forms of valuable needed support.

The association of loneliness with depressive symptomatology was found to vary by social network types. Only *diverse* network type (having the highest network size, the highest levels of support, below-average levels of conflict, and above-average levels of marital and friendship quality) and *family-focused* network type (having average network size, above-average support and the lowest levels of conflict from family, the highest marital quality but the lowest friendship quality) were shown to play a role in reducing the negative effect of loneliness on depressive symptomatology, which points to the potent effect of negative social exchanges and indicates that interpersonal conflicts and tensions could exacerbate the detrimental effects of precursors such as loneliness and stressors on depressive symptomatology. Future research should strive to elucidate the processes through which interpersonal conflicts and tensions and multiple precursors jointly influence depressive symptomatology. Specifically, interesting research to examine in the future is the effect of personality and stress in the features of social relationships, and their joint effect on depressive symptoms. It can be assumed that those who have personality disorder or experience accumulated stress could be more likely to belong to network types characterized by high levels of conflict and tension, or restricted network types, all of which can contribute to higher levels of depressive symptomatology (83–85).

The current study has several limitations that should be acknowledged and addressed in future research: First, because this study was based on cross-sectional data, the directionality among the research constructs remains uncertain. For instance, it is reasonable that individuals’ depressive symptoms may affect the levels of loneliness and the features of social relationships (e.g., network size, contact frequency, engagement in social activities, perceived levels of support, and relationship satisfaction) (86–88). Moreover, there are potential other confounders that we could not consider in our research model, such as major or minor accumulated life stressors, which could affect the pattern of results. Although we assume that the characteristics of network types have significant implications on depressive symptoms, bi-directional associations should be examined using a longitudinal dataset. In particular, changes in the attributes of individuals’ social network types with age and the progression of depressive symptoms deserve further research given the situational and dynamic nature of individuals’ social networks over the life course (44). Second, there are several other types of social relationships that could be considered such as neighbors, colleagues, distant family, and institutional ties. Including a more diverse range of both close and peripheral social ties could produce a more accurate portrait of individuals’ social network types and their implications on depressive symptomatology. Third, a more comprehensive set of covariates for depression other than loneliness and self-rated health (e.g., major life events, personality, chronic disease, and cognition) should be included to better understand the processes through which network types and multiple precursors jointly affect depressive symptoms. Fourth, although our use of a stratified probability sampling has the advantage of permitting generalizations of the observed associations to the middle-aged and older adult population, clinical patients and clinical measures of depression were not considered in this study. Future research should specifically recruit clinical sample and incorporate clinical measures of depression (e.g., BDI-II, GDS) (89, 90) or clinical diagnoses to examine the link between attributes of social networks, loneliness, and major depression. Lastly, it should be noted that this data was collected between 29 January and 5 February 2021, during which the COVID-19 pandemic was being controlled. Complying with social distancing guidelines could affect the levels and features of social interaction (especially among friends) and may lead to higher levels of loneliness and depressive symptoms among adults. The fact that the derived network types and their link with depressive symptomatology were broadly consistent with previous evidence (38, 39, 74) lends credence to the pattern of our results, but future studies should identify social network types and examine their associations with depressive symptomatology in a different context and replicate our results.

Despite these limitations, the current study of social network typologies and depressive symptomatology contributes to the literature by conceptualizing individuals’ social networks in a multidimensional construct and investigating the effects of network type membership on depressive symptomatology. Identifying social network types, as opposed to a variable-centered linear approach, provides a useful lens to understand which different combinations of social relationship attributes characterize individuals’ social networks, and how these heterotypic network types are associated with depressive symptoms. Taken together, our findings demonstrate that having multiple sources of supportive relationships and absence of conflicts and tensions are directly associated with lower depressive symptoms, and could also help buffer against the adverse effects of loneliness on depressive symptomatology. The findings based on network typologies could have important practical implications. Our profile-based approach identified middle-aged and older adults with both higher loneliness and worse health conditions as well as more limited social network resources as being at risk for severe depressive symptoms. Based on the results, health promotion interventions may aim to address various aspects of individuals’ social networks by providing support through the development of interpersonal skills, enhancing social integration within their existing social networks, decreasing exposure to conflicting social interactions, and strengthening the broader network resources.

## Data availability statement

The original contributions presented in the study are included in the article/Supplementary material, further inquiries can be directed to the corresponding author.

## Ethics statement

The studies involving human participants were reviewed and approved by Jeonbuk National University’s Institutional Review Board. The patients/participants provided their written informed consent to participate in this study.

## Author contributions

HS conceived of the study, helped analyses and interpretation of the data, and drafted the manuscript. CP did analyses and interpreted the data. All authors contributed to the article and approved the submitted version.

## Funding

The research received funding from the Brain Korea 21 fourth project of the Korea Research Foundation (Jeonbuk National University, Psychology Department no. 4199990714213).

## Conflict of interest

The authors declare that the research was conducted in the absence of any commercial or financial relationships that could be construed as a potential conflict of interest.

## Publisher’s note

All claims expressed in this article are solely those of the authors and do not necessarily represent those of their affiliated organizations, or those of the publisher, the editors and the reviewers. Any product that may be evaluated in this article, or claim that may be made by its manufacturer, is not guaranteed or endorsed by the publisher.

## Supplementary material

The Supplementary material for this article can be found online at: https://www.frontiersin.org/articles/10.3389/fpsyt.2023.1141370/full#supplementary-material

## Footnotes

^1^Researchers have often used a three-step approach in which they first build the latent profile model without distal outcomes, then determine the profile memberships, and subsequently investigate the relationship between profile memberships and distal outcomes using a simple ANOVA (66), whereas the BCH method uses a weighted multiple group analysis, where the groups correspond to the latent profiles, and thus is more robust against violations of assumptions (67).
