# Supplementary material for: Social network typologies moderate the association of loneliness with depressive symptomatology in middle-aged and older adults
Source: Front Psychiatry. 2023 May 5;14:1141370. doi: 10.3389/fpsyt.2023.1141370 (PMC10198616; doi:10.3389/fpsyt.2023.1141370)
Supplement: Supplementary file 1 [file Table_1.docx]

Appendix

Table A.1.

*Demographic information of participants*

|  | 40s | 50s | 60s | All |
| --- | --- | --- | --- | --- |
|  |  |  |  |  |
| Sample size (*N*) | 204 | 209 | 207 | 620 |
| Age, *mean (SD)* | 43.97 (2.91) | 53.20 (2.70) | 63.27 (2.73) | 53.52 (8.34) |
| Gender |  |  |  |  |
| Male, *N* (*%*) | 104 (33%) | 105 (33%) | 106 (34%) | 315 |
| Female, *N* (*%*) | 100 (33%) | 104 (34%) | 101 (33%) | 305 |
| Education |  |  |  |  |
| <High school, *N* (*%*) | 37 (26%) | 47 (32%) | 61 (42%) | 145 |
| Some college, *N* (*%*) | 149 (37%) | 133 (33%) | 120 (30%) | 402 |
| Graduate school, *N* (*%*) | 18 (25%) | 29 (40%) | 26 (36%) | 73 |
| Income |  |  |  |  |
| <$10,000, *N* (*%*) | 33 (23%) | 56 (40%) | 52 (37%) | 141 |
| $10,000–$20,000, *N* (*%*) | 15 (28%) | 14 (26%) | 25 (46%) | 54 |
| $20,000–$30,000, *N* (*%*) | 24 (30%) | 27 (34%) | 28 (35%) | 79 |
| $30,000–$40,000, *N* (*%*) | 34 (37%) | 21 (23%) | 38 (41%) | 93 |
| >$40,000, *N* (*%*) | 98 (39%) | 91 (36%) | 64 (25%) | 253 |
| Retirement status |  |  |  |  |
| Retired, *N* (*%*) | 11 (7%) | 26 (16%) | 125 (77%) | 162 |
| Not retired, *N* (*%*) | 193 (42%) | 183 (40%) | 82 (18%) | 458 |
| Marital status |  |  |  |  |
| Married, *N* (*%*) | 145 (30%) | 171 (35%) | 172 (35%) | 488 |
| Single, *N* (*%*) | 59 (45%) | 38 (29%) | 35 (27%) | 132 |
| Children |  |  |  |  |
| One or more, *N* (*%*) | 142 (27%) | 183 (35%) | 195 (38%) | 520 |
| None, *N* (*%*) | 62 (62%) | 26 (26%) | 12 (12%) | 100 |
|  |  |  |  |  |

Table A.2

*Fit indices and profile distributions for different latent profile solutions*

|  | 2 Profiles | 3 Profiles | 4 Profiles | 5 Profiles |
| --- | --- | --- | --- | --- |
| AIC | 31405.35 | 30626.46 | 30268.40 | 29932.05 |
| BIC | 31675.56 | 30989.69 | 30724.66 | 30481.34 |
| SABIC | 31481.90 | 30729.36 | 30397.65 | 30087.66 |
| Entropy | 0.88 | 0.91 | 0.89 | 0.89 |
| LMR | *p* < 0.01 | *p* < 0.01 | *p* = 0.17 | *p* = 0.75 |
| Profile composition (% based on estimated model) | | | | |
| Profile 1 | 69.03 | 56.13 | 48.55 | 40.81 |
| Profile 2 | 30.97 | 25.81 | 18.87 | 27.26 |
| Profile 3 |  | 18.07 | 17.58 | 14.68 |
| Profile 4 |  |  | 15.00 | 13.71 |
| Profile 5 |  |  |  | 3.55 |

*Note.* AIC = Akaike Information Criteria; BIC = Bayesian Information Criteria; SABIC = sample-size adjusted BIC; LMR-LRT = Lo-Mendell-Rubin likelihood ratio test; for AIC, BIC, and SABIC, smaller values indicate a better model. For entropy, higher values indicate a better classification quality (Celeux & Soromenho, 1996). Lo-Mendell-Rubin likelihood ratio test (LMR-LRT) is a measure of a relative fit where a significant *p* value implies that a model with k + 1 profiles is better than a model with k profiles (Lo et al., 2001; Nylund et al., 2007).

Table A.3

*Bivariate correlations and descriptive statistics for all research variables*

|  | *M (SD)* | 1 | 2 | 3 | 4 | 5 | 6 | 7 | 8 | 9 | 10 | 11 | 12 | 13 | 14 | 15 | 16 | 17 | 18 | 19 | 20 |  |
| --- | --- | --- | --- | --- | --- | --- | --- | --- | --- | --- | --- | --- | --- | --- | --- | --- | --- | --- | --- | --- | --- | --- |
| Network type variables | | | | | | | | | | | | | | | | | | | | | | |
| 1 | 78.70 | – |  |  |  |  |  |  |  |  |  |  |  |  |  |  |  |  |  |  |  |  |
| 2 | 3.40 (0.93) | .12^**^ | – |  |  |  |  |  |  |  |  |  |  |  |  |  |  |  |  |  |  |  |
| 3 | 2.65 (1.04) | -.02 | .48^**^ | – |  |  |  |  |  |  |  |  |  |  |  |  |  |  |  |  |  |  |
| 4 | 3.78 (1.54) | .05 | .41^**^ | .14^**^ | – |  |  |  |  |  |  |  |  |  |  |  |  |  |  |  |  |  |
| 5 | 2.86 (1.46) | -.06 | .23^**^ | .47^**^ | .25^**^ | – |  |  |  |  |  |  |  |  |  |  |  |  |  |  |  |  |
| 6 | 1.57 (0.90) | .49^**^ | .19^**^ | .07 | .06 | .02 | – |  |  |  |  |  |  |  |  |  |  |  |  |  |  |  |
| 7 | 1.54 (0.87) | .14^**^ | .16^**^ | .23^**^ | .08 | .14^**^ | .16^**^ | – |  |  |  |  |  |  |  |  |  |  |  |  |  |  |
| 8 | 3.65 (0.92) | .20^**^ | .43^**^ | .27^**^ | .30^**^ | .17^**^ | .13^**^ | .10^*^ | – |  |  |  |  |  |  |  |  |  |  |  |  |  |
| 9 | 3.22 (0.97) | .09^*^ | .33^**^ | .57^**^ | .12^**^ | .38^**^ | .11^**^ | .16^**^ | .57^**^ | – |  |  |  |  |  |  |  |  |  |  |  |  |
| 10 | 3.50 (0.96) | .16^**^ | .39^**^ | .38^**^ | .25^**^ | .24^**^ | .12^**^ | .12^**^ | .79^**^ | .71^**^ | – |  |  |  |  |  |  |  |  |  |  |  |
| 11 | 3.49 (0.96) | .01 | .23^**^ | .16^**^ | .05 | .02 | -.02 | .11^*^ | .46^**^ | .29^**^ | .42^**^ | – |  |  |  |  |  |  |  |  |  |  |
| 12 | 2.96 (0.83) | .03 | .19^**^ | .43^**^ | .05 | .37^**^ | .04 | .14^**^ | .23^**^ | .56^**^ | .30^**^ | .20^**^ | – |  |  |  |  |  |  |  |  |  |
| 13 | 2.83 (0.92) | .01 | .25^**^ | .18^**^ | .10^*^ | .09^*^ | .05 | .06 | .32^**^ | .27^**^ | .26^**^ | .29^**^ | .34^**^ | – |  |  |  |  |  |  |  |  |
| 14 | 2.66 (0.97) | .04 | .32^**^ | .19^**^ | .15^**^ | .04 | .01 | .02 | .33^**^ | .30^**^ | .23^**^ | .23^**^ | .43^**^ | .45^**^ | – |  |  |  |  |  |  |  |
| 15 | 2.42 (0.92) | -.03 | -.10^*^ | -.04 | -.10^*^ | -.01 | .03 | .06 | -.26^**^ | -.08 | -.24^**^ | -.37^**^ | .14^**^ | .11^*^ | .10^*^ | – |  |  |  |  |  |  |
| 16 | 1.80 (0.83) | -.01 | -.02 | .04 | -.12^**^ | .01 | .03 | .11^**^ | -.13^**^ | .02 | -.07 | .06 | .22^**^ | .17^**^ | .14^**^ | .41^**^ | – |  |  |  |  |  |
| 17 | 1.99 (0.86) | .10^*^ | -.07 | -.01 | -.03 | -.05 | .05 | .04 | -.18^**^ | -.05 | -.14^**^ | .01 | .14^**^ | .07 | .15^**^ | .45^**^ | .58^**^ | – |  |  |  |  |
| 18 | 1.76 (0.83) | -.06 | -.10^*^ | .01 | -.11^**^ | -.04 | -.02 | .07 | -.22^*^* | -.02 | -.14^**^ | -.03 | .16^**^ | .14^**^ | .08 | .35^**^ | .60^**^ | .57^**^ | – |  |  |  |
| 19 | 3.59 (1.02) | .03 | .26^**^ | .19^**^ | .05 | .04 | .01 | .08 | .49^**^ | .30^**^ | .48^**^ | .77^**^ | .10^*^ | .16^**^ | .13^**^ | -.52^**^ | -.06 | -.14^**^ | -.10^*^ | – |  |  |
| 20 | 2.61 (0.68) | -.01 | .08^*^ | .34^**^ | -.04 | .24^**^ | .01 | .09^*^ | .14^**^ | .46^**^ | .25^**^ | .17^**^ | .67^**^ | .34^**^ | .40^**^ | .19^**^ | .35^**^ | .25^**^ | .31^**^ | .07 | – |  |
| Demographic variables | | | | | | | | | | | | | | | | | | | | | | |
| 21 | 53.52 (8.34) | .14^**^ | .05 | -.01 | -.01 | .02 | .25^**^ | .11^**^ | .08 | .02 | .05 | .02 | -.02 | .15^**^ | -.03 | -.03 | -.07 | -.20^**^ | -.08 | .02 | -.02 |  |
| 22 | 49.19 | -.02 | .03 | -.08 | .12^**^ | .09^*^ | -.02 | -.06 | -.02 | -.04 | -.03 | -.12^*^ | .01 | .23^**^ | .04 | .07 | -.15^*^* | .03 | -.07 | -.23^*^* | -.12^**^ |  |
| 23 | 3.87 (0.63) | .13^**^ | .05 | .04 | .01 | -.07 | .01 | .14^**^ | .07 | .09^*^ | .10^*^ | .14^**^ | -.02 | -.09^*^ | -.02 | -.03 | .02 | .01 | -.01 | .16^**^ | .03 |  |
| 24 | 3.42 (1.61) | .17^**^ | .05 | .07 | .11^**^ | -.01 | .03 | .12^**^ | .10^**^ | .07 | .10^*^ | .14^**^ | .02 | -.04 | .09^*^ | -.03 | .09^*^ | .10* | .03 | .08 | .07 |  |
| 25 | 3.15 (0.76) | .13^**^ | .17^**^ | .16^**^ | .06 | .06 | .11^**^ | .14^**^ | .17^**^ | .18^**^ | .22^**^ | .07 | .10^*^ | .07 | .07 | .01 | -.01 | -.04 | -.06 | .13^**^ | .08 |  |
| Mental health | | | | | | | | | | | | | | | | | | | | | | |
| 26 | 2.56 (0.70) | -.13^*^* | -.41^**^ | -.49^**^ | -.23^**^ | -.36^**^ | -.12^**^ | -.18^*^ | -.57^**^ | -.65^**^ | -.63^**^ | -.29^**^ | -.40^**^ | -.23^**^ | -.26^**^ | .23^**^ | .18^**^ | .25^**^ | .24^**^ | -.35^**^ | -.24^**^ |  |
| 27 | 16.18 (11.33) | -.12^*^* | -.32^**^ | -.24^**^ | -.22^**^ | -.12^**^ | -.10^*^ | -.08^*^ | -.48^**^ | -.36^**^ | -.47^**^ | -.29^**^ | -.08^*^ | -.09^*^ | -.12^**^ | .35^**^ | .23^**^ | .26^**^ | .30^**^ | -.43^**^ | .02 |  |

*Note.* 1. Married (%) 2. Family size 3. Friend size 4. Family contact frequency 5. Friend contact frequency 6. Number of children 7. Number of social activities 8. Perceived support: family 9. Perceived support: friend 10. Perceived support: close others 11. Received support: spouse 12. Received support: friend 13. Received support: child 14. Received support: sibling 15. Spouse conflict 16. Friend conflict 17. Child conflict 18. Sibling support 19. Marital quality 20. Friendship quality 21. Age 22. Female (%) 23. Education 24. Income 25. Self-rated health 26. Loneliness 27. Depressive symptomatology. ^*^*p* < .05. ^**^*p* < .01.
